# An Aggressive Presentation of Mantle Cell Lymphoma With Unique Molecular Features

**DOI:** 10.7759/cureus.17598

**Published:** 2021-08-31

**Authors:** Arati A Inamdar, Abraham Loo, Nagy Mikhail, Patrick Lee

**Affiliations:** 1 Pathology, RWJBarnabas Health, Livingston, USA; 2 Pathology and Laboratory Medicine, Rutgers-Robert Wood Johnson Medical School, New Brunswick, USA; 3 Pathology, Rutgers-Robert Wood Johnson Medical School, New Brunswick, USA; 4 Hematology Oncology, Monmouth Medical Center, Long Branch, USA

**Keywords:** cyclin d, next generation sequencing, non hodgkin lymphoma, mantle cell lymphoma, molecular profile

## Abstract

Mantle cell lymphoma (MCL) is an aggressive non-Hodgkin lymphoma (NHL) with a dismal prognosis. The pathogenesis of MCL is complex and involves molecular alterations in various genes and pathways including the regulatory elements of the cell cycle machinery and senescence, DNA damage response pathways, and cell survival signals. Currently, Mantle Cell Lymphoma International Prognostic Index (MIPI) score and proliferative gene markers. *TP53* and *CDKN2A* alterations are being used for the prognosis of MCL patients. The molecular profiling performed with various expression studies has paved the way for the identification of novel molecular targets and novel biomarkers not only aid in the diagnosis and prognosis of MCL but also predict the clinical outcome and prognosis. Our patient is a 74-year-old male who came for urinary complaints and routine blood work and revealed leukocytosis and lymphocytosis with abdominal and pelvic lymphadenopathies. Further work-up confirmed the diagnosis of MCL involving peripheral blood, bone marrow, and colon. In our patient, due to aggressive presentation, next generation sequencing was performed to understand the genetic aberrations relevant for MCL. In addition to known markers, we identified genetic mutations in *FAT1*, *IKZF3,* and *TRAF2*. which have never been reported in MCL and could be pathogenic for the aggressive presentation of our patient and thus could be further investigated with in vitro and animal models.

## Introduction

Mantle cell lymphoma (MCL) is a lymphoid malignancy of the B-cells, developing from naïve pre-germinal center cells of primary follicles or mantle regions of secondary follicles. MCL is a rare, clinically aggressive, and incurable form of non-Hodgkin lymphoma (NHL) predominantly seen in older males as compared to females [1]. Patients with MCL account for 5% of NHL cases and often present with an advanced-stage disease with a mean survival of only three to four years [2]. The classic immunophenotype is characterized by coexpression of B-cell markers (CD19, CD20, and CD 43) and T cell-associated CD5+ marker, and absence of germinal center markers, CD10, CD23, and Bcl-6. The pathognomic feature of MCL is the chromosomal translocation t(11;14) (q13;q32) (IGH/CCND1), which is observed in >95% of cases, resulting in the constitutive overexpression of G1-phase cell cycle protein cyclin D1 (CCND1). Additional mutations also work in concert with CCND1 overexpression to exacerbate lymphomagenesis through cell cycle deregulation, potentially leading to more aggressive blastoid or pleomorphic MCL [3]. Although MCL is typically associated with the rapid evolution and aggressive behavior that is resistant to therapy, there exists a subset of MCL cases that follow an indolent and stable clinical course. Such patients present with a non-nodal disease, hypermutated IgVH, and absence of SOX11 expression [4].

More recently, next generation sequencing (NGS) is being used to identify the key mutations in MCL, and published reports have utilized comprehensive whole-genome/whole-exome analysis of patients’ samples to identify the common signature genes associated with MCL [5]. Such mutations may serve as a guide for risk assessment, screening, differential diagnosis, determination of prognosis, prediction of response to treatment, and monitoring progression of the disease. The comprehensive list of key biomarkers for MCL has been reviewed by Inamdar et al. 2016 [2]. 

We present a unique case of a 74-year-old male with a past medical history of hypertension and atrial fibrillation who presented to the emergency department with symptoms of urinary obstruction and persistent watery diarrhea without blood or mucus for the past three weeks. The laboratory work-up and imaging revealed leukocytosis and lymphocytosis with abdominal and pelvic lymphadenopathies. The flow cytometry and immunostaining confirmed the diagnosis of mantle cell lymphoma extending to the large bowel. The molecular testing was performed on bone marrow aspirate to reveal six identified mutations in key genes, three that have never been previously reported in any published cases of mantle cell lymphoma. The patient was started on bendamustine-rituximab and demonstrated complete remission after six cycles.

## Case presentation

**Figure 1:**
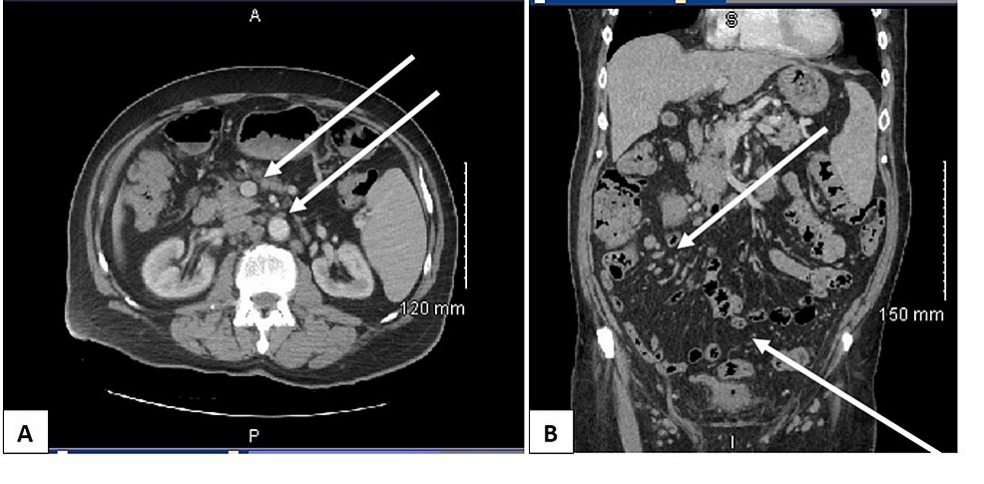
CT scan demonstrating the diffuse pelvic lymphadenopathy. The transverse (A) and coronal (B) planes with enlarged pelvic lymph nodes (arrows).

We present a unique case of a 74-year-old male with a past medical history significant of hypertension and atrial fibrillation who presented to the emergency department with symptoms of urinary obstruction and persistent watery diarrhea without blood or mucus for the past three weeks. The patient denied any fever, shortness of breath, or night sweats. The physical examination revealed splenomegaly without cervical, axillary, or inguinal lymphadenopathy. The routine complete blood count (CBC) revealed leukocytosis and lymphocytosis of 29,000/ul and 24,000/ul, respectively. The hemoglobin, hematocrit, and platelets were within normal limits. The CT scan of the abdomen/pelvis revealed splenomegaly (17cm) and adenopathy of the pericaval (1.5cm), para-aortic (2.6cm), mesenteric right (4cm), and both right and left external iliac arteries (Figure 1 A, B).

**Figure 2:**
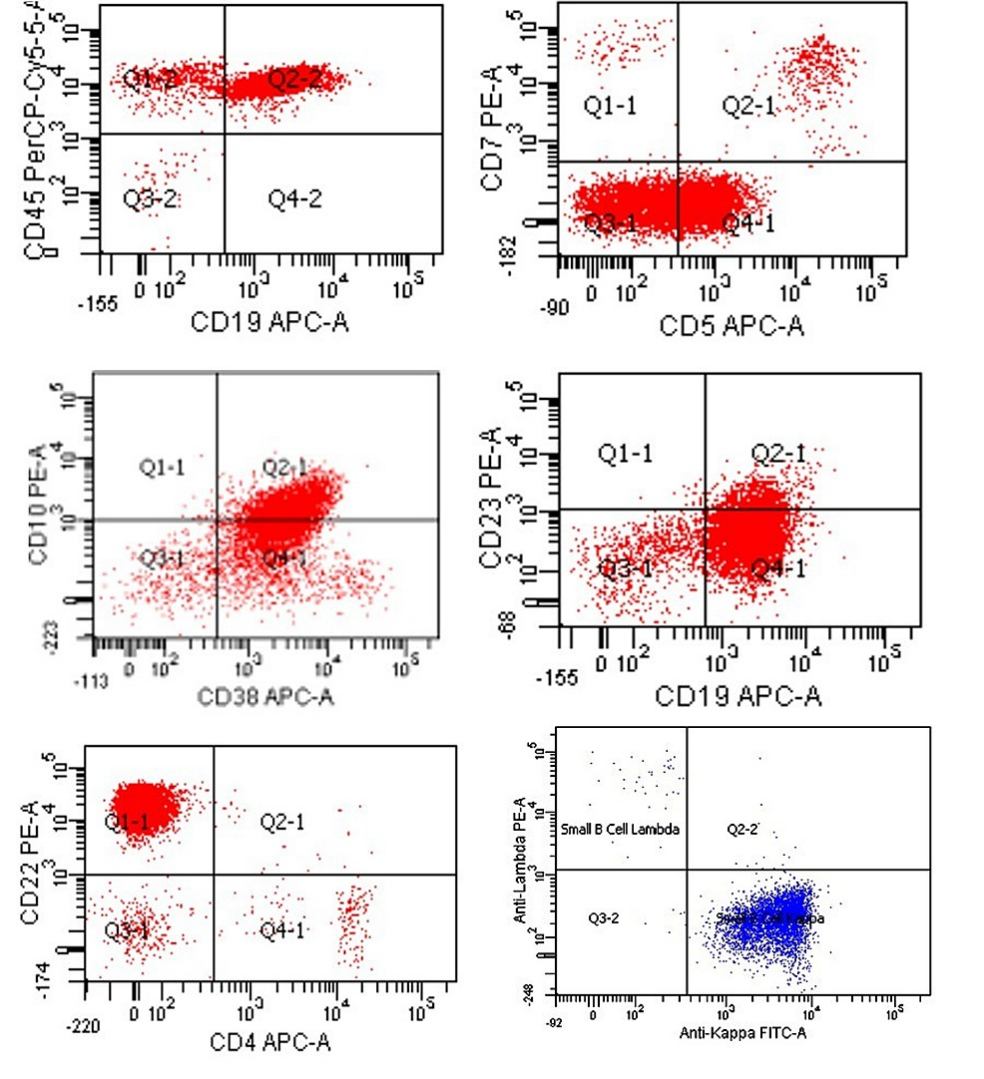
Flow cytometry analysis of peripheral blood. The peripheral blood showed monoclonal surface kappa positive population with positive expression for CD19, CD 45, CD5 (dim to negative), CD10 (dim), CD38, CD22, and CD 23 (dim).

The peripheral blood was sent for flow cytometry analysis. The analysis detected small and large gates comprising 53% and 4% of total events, respectively. Within the small and large cell gates, 77% and 70% cells respectively were monoclonal surface light chain kappa positive population expressing CD45, CD10 (dim), CD38, CD22, and dim CD5 (Figure 2).

**Figure 3:**
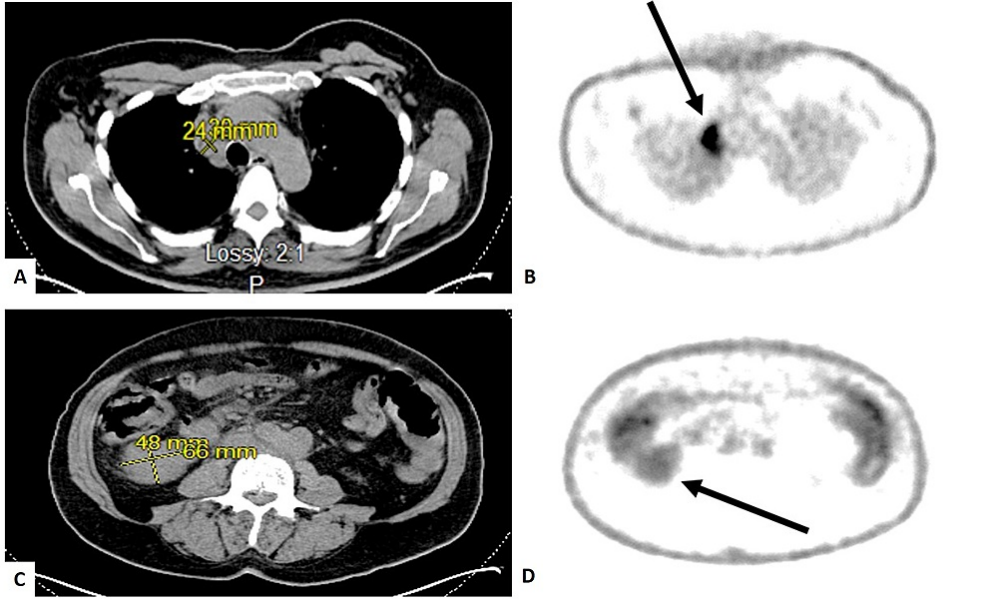
CT and corresponding PET scan demonstrating the PET avidity (arrows) in the enlarged lymph nodes. The transverse plane demonstrates PET avid enlarged mediastinal lymph node (A, B) and lymph node in the right lower quadrant (C, D). PET: positron emission tomography

The positron emission tomography (PET) scan revealed lymphadenopathy in the right supraclavicular region, mediastinum, bilateral axilla, retroperitoneal, and mesenteric regions along with hypermetabolic mass with SUV of 7.0 at the right lower quadrant measuring 4.8 x 6.6 x 2.4 cm (Figure 3 A-D). 

**Figure 4:**
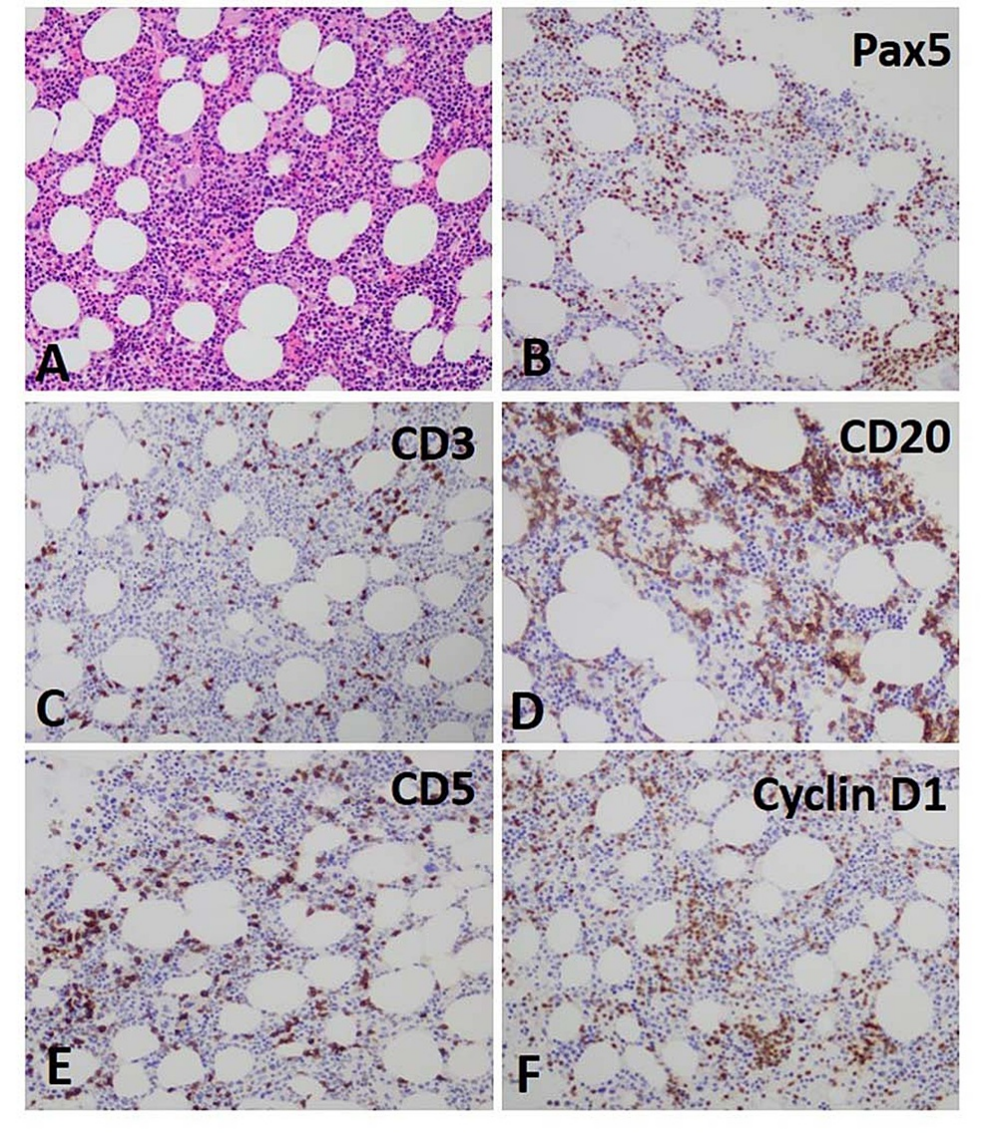
Bone marrow biopsy Hematoxylin and eosin highlight the small lymphocytes (A). The immunostaining demonstrates the increased Pax 5, CD20 and presence of cyclin D1 positive cells along with kappa restriction (B, D, and F, respectively). Mildly increased CD5 positive cells with normal CD3 and lambda expression are also noted (C and E, respectively).

The bone marrow biopsy was performed for staging of mantle cell lymphoma. The normocellular (40%) bone marrow showed a normal myeloid/erythroid ratio with 40-60% atypical lymphocytes seen as small aggregates and demonstrate co-expression for CD20/PAX5/Cyclin D1 (strong) (Figure 4 A-F).

**Figure 5:**
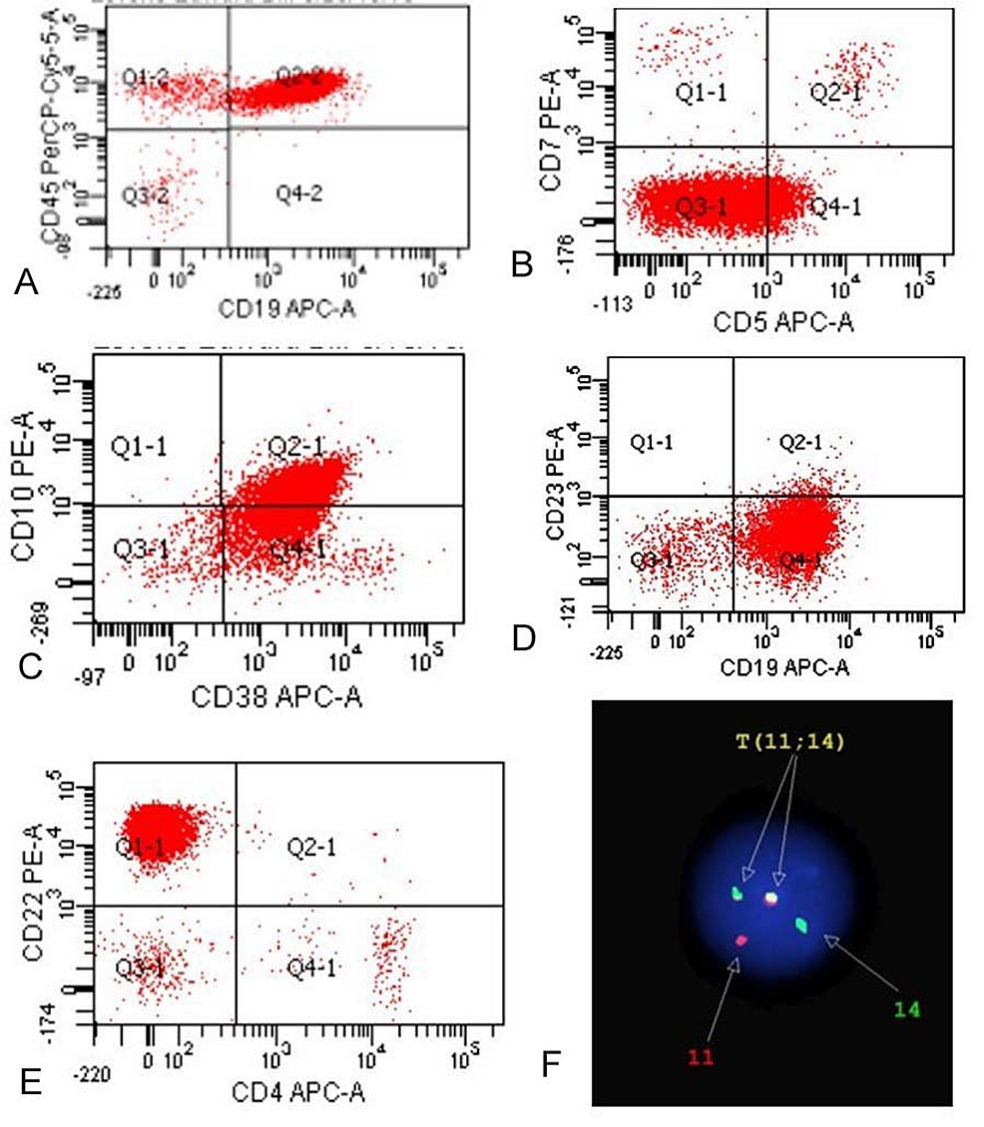
Flow cytometry and FISH studies on bone marrow Monoclonal surface kappa positive population shown with positive expression for CD19, CD 45, CD5 (dim to negative), CD10 (dim), CD38, CD22, and CD 23 (dim). The FISH study revealed translocation (11; 14) confirming the diagnosis for MCL. FISH: fluorescent in situ hybridization

The flow cytometry analysis showed small and large cell gates comprising 67% and 12% of total events, respectively. Within both gates, monoclonal surface kappa positive population was identified with positive expression for CD20 (bright), CD19, CD5 (dim to negative), CD45, CD10 (dim), CD38, and CD22 and comprised of 72% of total events (Figure 5 A-E). Chromosome analysis of bone marrow reveals a 46, XY male karyotype. FISH study for t(11;14) IGH/CCND1 gene rearrangement revealed abnormal signal pattern in 13% interphase cells consistent with IGH/CCND1 gene rearrangement using Vysis LSI IGH/CCND1 dual color dual fusion probes (Figure 5 F).

**Figure 6:**
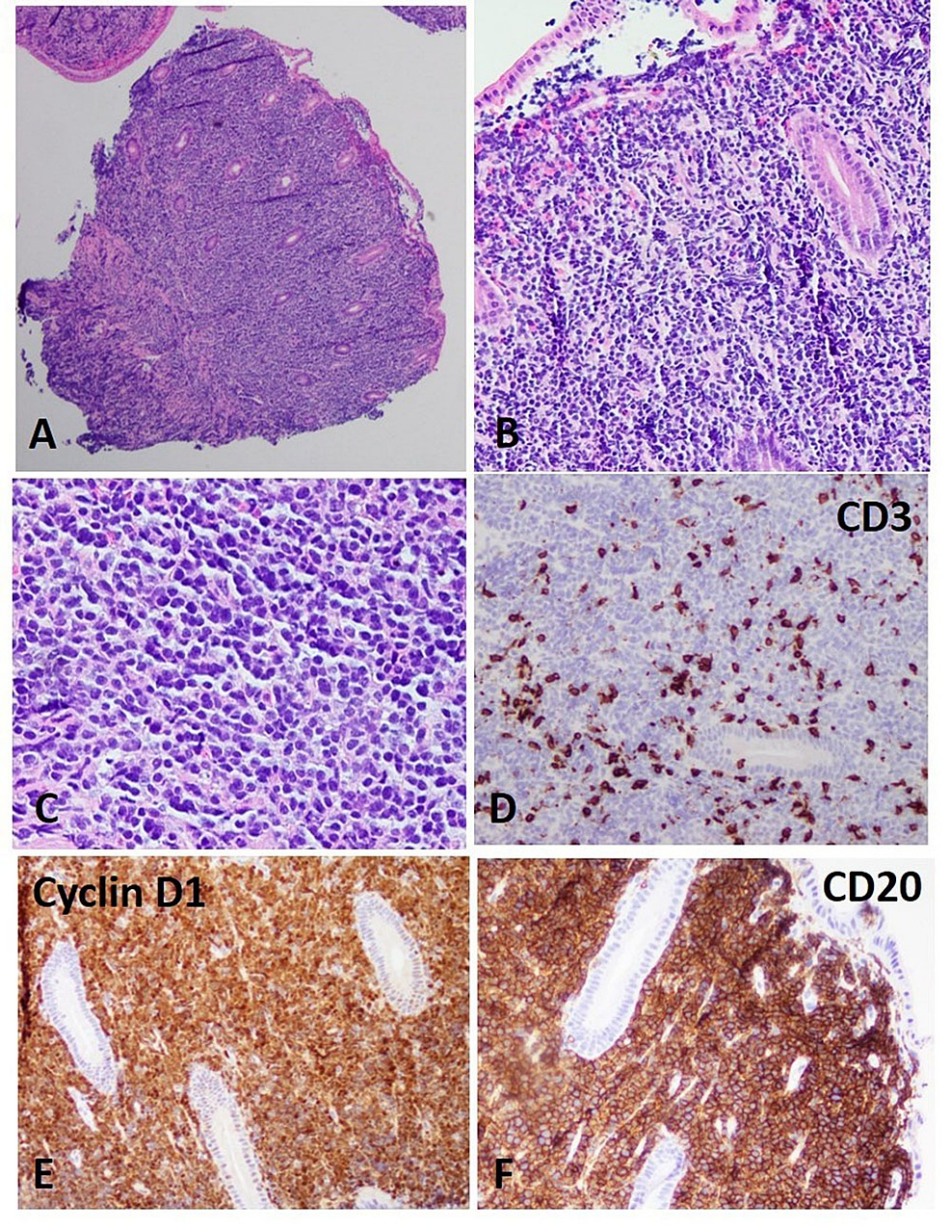
Colonoscopy of large bowel The biopsy of the large bowel demonstrated atypical small to medium size lymphocytes (A-D) in the lamina propria, which were diffusely positive for CD20 and cyclin D1 (E and F, respectively). Few scattered CD3 positive lymphocytes were also identified (D).

The colonoscopy revealed mucosal thickening of the right, left, and sigmoid colon as well as rectum. The representative biopsy of these areas demonstrated atypical small to medium size lymphocytes in the lamina propria. The atypical lymphocytes are strongly and diffusely positive for CD20 and cyclin D1 consistent with Mantle cell lymphoma (Figure 6 A-F).

The DNA from bone marrow cells from aspirate was subjected to a customized Next Generation Sequencing panel to identify single nucleotide variants (SNV), copy number variants (CNV), and insertion/deletion covering 237 reportable genes (Lymphoid panel, Genoptix, CA, USA). Quality control metrics include a minimum input of 20 ng, with an optimal input of 100ng of genomic DNA, and an average mean sequencing depth of 500x coverage. The genomic alterations within each of these genes are detected through proprietary bioinformatics analysis software and interpreted in conjunction with reference databases such as the Catalogue Of Somatic Mutations In Cancer (COSMIC) and the single nucleotide polymorphism database (dbSNP). Mutation in six genes was identified along with their allele frequency and pathogenicity are listed in Table 1. Of these, *ATM*, *MEF2B*, and *TP53* have been previously reported in cases of mantle cell lymphoma but the genetic mutations in *FAT1*, *IKZF3,* and *TRAF2* were novel to our case of mantle cell lymphoma.

**Table 1:** The details of the mutation along with their allele frequency and pathogenicity in six genes with next generation analysis using bone marrow aspirate.

Gene	Exon Tested	Genomic Alteration (8)	Mutation Effect	Allele Frequency	Pathogenic
ATM	ALL	c.3454delT: p.S1152Lfs’4	FRAMESHIFT	20%	YES
FAT1	ALL	c.10268C˃T: p.T3423M	MISSENSE	48%	UNCERTAIN
I*KZF3*	ALL	c.71C˃T: p.A24V	MISSENSE	49%	UNCERTAIN
MEF2B	2-8	c.68A˃G: p.K23R	MISSENSE	38%	LIKELY
TP53	ALL	c.945_946delTC: p.Q317Af5’19	FRAMESHIFT	54%	YES
TRAF2	ALL	c.683_684delAG: p.E228Gf5’2	FRAMESHIFT	49%	YES

## Discussion

MCL, being an aggressive disease, remains a challenging prospect despite advances in the development of clinical agents for its treatment. It can present with either an indolent or aggressive course; aggressive MCL usually presents with high LDH and MIPI score and has poor prognosis. In our patient, with MIPI score of 6.5 and involvement of colon and rectum, aggressive nature of MCL disease was evident (MIPI score > 6.2, and 5.7-6.2 indicate high and low disease risk, respectively).

MCL commonly involves lymph nodes but the involvement of extra-nodal sites such as the gastrointestinal tract, Waldeyer ring, spleen, and bone marrow with or without peripheral blood involvement are also seen in advance stages [6]. The flow cytometry analysis of MCL typically includes positive reactivity for CD19/CD20/CD5/FMC7 with the bright expression of surface immunoglobulins and negative reactivity for CD10/CD23 [2]. However, variations in immune-phenotype such as positive CD10/CD23 or negative CD5 have also been reported in some MCL cases [7]. In our patient, flow cytometric analysis of bone marrow aspirate and peripheral blood demonstrated monoclonal surface kappa positive population with positive expression for CD20 (bright), CD19, CD5 (dim to negative), CD45, CD10 (dim), CD38 and CD22 suggestive of the immunophenotypic aberrancies (Figure 2 and Figure 4). Although expression of such aberrant markers appears to be devoid of any significant effect on progression-free or overall survival, more studies are needed to further substantiate the observed relationship.

In recent years, molecular studies have advanced our knowledge in understanding the disease pathogenesis as well as for the identification of molecular targets for early diagnosis and prognosis. Somatic mutations are known to alter the normal cell function, which leads to cancer initiation and progression. More recently epigenetic events especially those involved with chromatin modification and DNA methylation are seen as crucial in cancer progression [8]. Several studies have identified mutations in the somatic and epigenetic genes using whole genome sequencing (WGS), whole exome sequencing (WES), and micro RNA (miRNA) expression profiling [9]. Similar techniques have been implemented to report somatic and epigenetic mutations seen in MCL patients [8]. Such studies not only identify common molecular aberrations in both newly diagnosed as well as relapse/recurrent MCL patients but also determine the prognostic and therapeutic implications of such molecular targets taking into consideration conventional and non-nodal leukemic types of MCL [10]. 

The common somatic and epigenetic mutations identified in MCL include ATM, CCND1, TP53, MLL2, MLL3, TRAF2, NOTCH1, NOTCH2, WHSC1, BIRC3 and UBR5. Of these, ATM, CCND1 and TP53 occur in higher (usually >40%) frequency [11]. Other studies have identified previously unreported mutations namely CCDC15, APC, CDH1, S1PR1, ATRX, BRCA2, CASP8, and NOTCH3 [5]. In our patient, we identified the mutations in six genes namely *ATM, FAT1, IKZF3, MEF2B, TP53* and *TRAF2* (Table 1).

*ATM*, a tumor suppressor gene located on 11q22-q23, encodes for a cell cycle checkpoint kinase belonging to the phospho-inositol-3/phospho-inositol-4 kinase family [2]. *ATM* mutations are also in 40-75% of MCL with one study demonstrating the deleterious effect of *ATM* mutation occurring from premature termination of translation and subsequent absence of the phospho-inositol kinase 3-domain [12]. Some patients demonstrated mutations equivalent to disease mutations associated with T-prolymphocytic leukemia or causative for ataxia-telangiectasia [13]. *ATM* mutations also are found in 12% of chronic lymphocytic leukemia, and are associated with unmutated *IGHV*, 11q deletion, and an unfavorable prognosis. A recent report indicated that mutations in *ATM* are devoid of any prognostic value. Of note, *ATM* mutations are associated with SOX11-positive tumors, whereas *CCND1* mutations are detected in MCL with IgVH-mutations [4]. Furthermore, *ATM* mutation/deletion leads to a radio-sensitization of the tumor in animal model suggesting that radiotherapy could be one of the therapeutic approaches for MCL with *ATM* deletion [14]. The frameshift mutation (c.34S4delT; p.S1152Lfs"4) seen in our patient has never been reported previously but appears to be pathogenic.

*FAT1*, located on 4q35, encodes for a tumor suppressor protein encoding for a cadherin-like protein, essential for controlling cell proliferation during development [15]. *FAT1* mutation has never been previously reported in MCL patients. The mutations in *FAT1* gene have been shown to negatively regulate WNT signaling, and are found in 10% of fludarabine refractory-small lymphocytic leukemia as well as in untreated chronic lymphocytic leukemia, suggesting a role in the development of a high-risk phenotype [16]. *FAT1* mutations are also found in 25% of adult early T-cell precursor acute lymphoblastic leukemia, 12% of adult T-cell acute lymphoblastic leukemia, and 7% of splenic marginal zone lymphoma. Interestingly, *FAT1* provides an independent prognostic factor for relapse-free and overall survival in pediatric pre-B-cell-acute lymphoblastic leukemia (pre-B-ALL) [17]. A genomic alteration causing missense mutation in the *FAT1* gene is detected (c.10268C>T; p.T3423M) in our patient and its pathogenicity appears to be uncertain due to lack of enough data.

*IKZF3*, located on 17q12-q21.1, encodes for a member of the Ikaros family of zinc-finger proteins, which are involved in the regulation of B-cell differentiation, proliferation, and maturation to an effector state. It is also involved in regulating *BCL2* expression and controlling apoptosis in T-cells in an *IL2*-dependent manner [18]. *IKZF3* mutations are found in 13% of near-haploid pediatric acute lymphoblastic leukemia and 2% of chronic lymphocytic leukemia; however, *IKZF3* mutations have never been reported in MCL. In our patient, genomic alteration in the *IKZF3* gene is detected (c.71C>T; p.A24V). This missense alteration has been previously reported both in samples of endometrial carcinoma and hemangioblastoma and as a germline variant in 99 apparently normal individuals of various ethnicities [18]. Based on the available evidence, its clinical significance is uncertain.

*MEF2B*, located on 19p13.11, encodes for a member of the MADS/MEF2 family of the DNA binding proteins, which are thought to regulate gene expression, including expression of the smooth muscle myosin heavy chain gene [18]. *MEF2B* mutations found in 18% of diffuse large B-cell lymphoma; 10% of follicular lymphoma, where they are associated with a more favorable prognosis and 3% of MCL [5]. A genomic alteration in the *MEF2B* gene is detected in our patient (c.68A>G; p.K23R). This missense variant has been previously reported in MCL and has been shown to reduce DNA-binding ability (FWD; 9111360) and is therefore expected to be likely pathogenic.

*TP53*, located on 17p13.1, encodes for a tumor suppressor protein that responds to diverse cellular stresses by regulating expression of target genes, thereby inducing cell cycle arrest, apoptosis, senescence, DNA repair, or changes in metabolism [2, 18]. Somatic *TP53* alterations are frequent in most human cancers, and germline *TP53* mutations predispose to a wide spectrum of early-onset cancers including Li-Fraumeni and Li-Fraumeni-like syndromes. *TP53* alterations including *TP53* mutations or 17p deletions are associated with an unfavorable prognosis and low response rates to chemotherapy and immunotherapy in chronic lymphocytic leukemia, and patients with *TP53* alterations may potentially benefit from agents including BTK, BCL2, and P13K delta inhibitors [19]. *P53* alterations are also found in a variety of other lymphoid neoplasms, and are typically associated with an unfavorable prognosis. A genomic alteration in the *TP53* gene is detected (c.945_946deITC; p.0317Afs'19). This frameshift alteration has been previously reported and is expected to be pathogenic.

*TRAF2*, located on 9q34, encodes for a protein that is required for TNF-alpha-mediated activation of MAPK8/JNK and NF- kappa. The protein complex formed by *TRAF2* and *TRAF1* interacts with the inhibitors of apoptosis (IAP) family members, cIAP1 and cIAP2, and functions as a mediator of the anti-apoptotic signals from TNF receptors. *TRAF2* mutations are found in 6% of mantle cell lymphoma, 3% of Waldenstrom macroglobulinemia and 1% of chronic lymphocytic leukemia [18]. *TRAF2* mutations via involvement of the alternative NF-kappa pathway may be associated with resistance to *SCR* inhibition [20]. A genomic alteration in the *TRAF2* gene is detected (c.683_684delAG; p.E229Gfs*2). This frameshift alteration has not been previously reported to our knowledge in MCL, but is expected to be pathogenic.

## Conclusions

In conclusion, we present a rare scenario of an aggressive presentation of MCL involving the extranodal colonic mucosa and peripheral blood and bone marrow. The patient showed complete remission with six cycles of rituximab-bendamustine. In addition to previously reported genetic mutations in *ATM, TP53*, and *MEF2B*, our patient also demonstrated novel mutations in *FAT1, IKZF3, *and *TRAF2*, which have never been reported in MCL. These findings indicate the pathogenic significance of such novel genes for MCL, especially for the aggressive form of extra-nodal presentation of MCL, and could be worth further investigating.
